# A facile synthesis of a carbon-encapsulated Fe_3_O_4_ nanocomposite and its performance as anode in lithium-ion batteries

**DOI:** 10.3762/bjnano.4.79

**Published:** 2013-10-30

**Authors:** Raju Prakash, Katharina Fanselau, Shuhua Ren, Tapan Kumar Mandal, Christian Kübel, Horst Hahn, Maximilian Fichtner

**Affiliations:** 1 Institute for Nanotechnology (INT), Karlsruhe Insititute of Technology (KIT), Hermann-von-Helmholtz-Platz 1, Karlsruhe, 76344, Germany; 2 current address: Centre for Automotive Energy Materials (CAEM), International Advanced Research Centre for Powder Metallurgy and New Materials (ARCI), Taramani, Chennai-600113, India; 3 Faculty of Science and Technology, ICFAI University, Selaqui, Dehradun-248197, India; 4 Karlsruhe Nano Micro Facility (KNMF), Karlsruhe Institute of Technology (KIT), Hermann-von-Helmholtz-Platz 1, Karlsruhe, 76344, Germany; 5 Helmholtz Institute Ulm (HIU), Albert-Einstein-Allee 11, Ulm, 89081, Germany

**Keywords:** electrochemistry, iron oxide, lithium-ion battery, nanoparticles, pyrolysis

## Abstract

A carbon-encapsulated Fe_3_O_4_ nanocomposite was prepared by a simple one-step pyrolysis of iron pentacarbonyl without using any templates, solvents or surfactants. The structure and morphology of the nanocomposite was investigated by X-ray diffraction, scanning electron microscopy, transmission electron microscopy, Brunauer–Emmett–Teller analysis and Raman spectroscopy. Fe_3_O_4_ nanoparticles are dispersed intimately in a carbon framework. The nanocomposite exhibits well constructed core–shell and nanotube structures, with Fe_3_O_4_ cores and graphitic shells/tubes. The as-synthesized material could be used directly as anode in a lithium-ion cell and demonstrated a stable capacity, and good cyclic and rate performances.

## Findings

Due to high energy density and excellent cyclic performance, lithium-ion batteries (LIBs) have become the leading energy storage device for portable electronic markets and for powering upcoming electric vehicles [1–2]. In order to obtain LIBs with superior performance, numerous strategies to find new materials are currently being explored [3]. Fe_3_O_4_ is widely regarded as one of the high energy-density anode materials for LIBs, and is based on the conversion mechanism (Fe_3_O_4_ + 8 Li^+^ + 8 e^−^ ↔ 3 Fe + 4 Li_2_O) [4–6]. The theoretical specific capacity of Fe_3_O_4_ is 926 mAh·g^−1^, which is far beyond that of a graphite anode (372 mAh·g^−1^). However, because of agglomerations and the significant volume change of active materials during the redox reaction, Fe_3_O_4_ anodes have suffered greatly from poor cyclic performances. A variety of strategies, such as carbon coatings [7], carbon core–shells [8], nanocomposites [9], nanostructures [10], or nano-encapsulation [11], have recently been explored to circumvent this problem. These strategies apply various synthetic methods [12] such as hydrothermal, coprecipitation, microemulsion, sol–gel, plasma synthesis, electro-spray, and laser pyrolysis techniques. Much improved electrochemical performances have been achieved with the modified materials [13].

However, all aforementioned methods need multi-step processes that include removing solvents, surfactants, or templates. Especially, the removal of solvents deposited on the nano-Fe_3_O_4_ surfaces is a major challenge, which restricts their practical applications [12]. Hence, it is crucial to develop a straightforward and solvent-free process for the synthesis of Fe_3_O_4_ nanocomposite.

Herein, we report a simple method that directly affords a carbon encapsulated Fe_3_O_4_ nanocomposite [Fe_3_O_4_–C] by employing Fe(CO)_5_ precursor without any templates, solvents, or surfactants. This raw material acts not only as the source of iron and oxygen, but also of carbon, which gives rise to typical nanostructures. Fe_3_O_4_ nanoparticles are dispersed intimately in a carbon framework. The material could be used directly as anode and yielded a stable capacity.

In a typical synthesis, Fe(CO)_5_ was sealed into a closed stainless steel Swagelok-type reactor under argon atmosphere as described previously [14]. The reactor was placed horizontally inside the home made rotating quartz-tube setup [15] in a furnace. The tube was rotated at 10 rpm during pyrolysis to obtain a homogeneous mixture. The reactor was heated at a rate of 5 °C·min^−1^ to 700 °C and kept at this temperature for 3 h. The reaction took place under autogenous pressure. After allowing the reactor to cool down to room temperature, the remaining pressure was released carefully. A dry fine black powder of [Fe_3_O_4_–C] produced was collected and used directly without any further treatment. The reaction precedes in two steps: in the first step, Fe(CO)_5_ decomposes to form Fe and CO gas {Fe(CO)_5_(g) → Fe(s) + 5CO(g)} [16]. Subsequently, CO reacts with the active Fe nanoparticles to yield Fe_3_O_4_ nanoparticles and carbon {Fe(s) + CO(g) → Fe_3_O_4_–C*_x_*(s) + gaseous material}. The iron nanoparticles catalyze the formation of nanotubes and shells from the in-situ generated carbon. Meanwhile, the Fe_3_O_4_ produced from the Fe nanoparticles is encapsulated within the nanotubes or carbon shells. Elemental analysis suggested that the composite consists of 70 wt % of Fe_3_O_4_ and 30 wt % of carbon. The energy dispersive X-ray spectroscopy (EDX) patterns by using SEM mode (Figure S1 in Supporting Information File 1) show that the as prepared [Fe_3_O_4_–C] is composed of C, Fe and O. The observed Fe/O mass ratio of the composite (ca. 2.7) is in close agreement with the nominal value of Fe_3_O_4_ (2.62).

**Figure 1:**
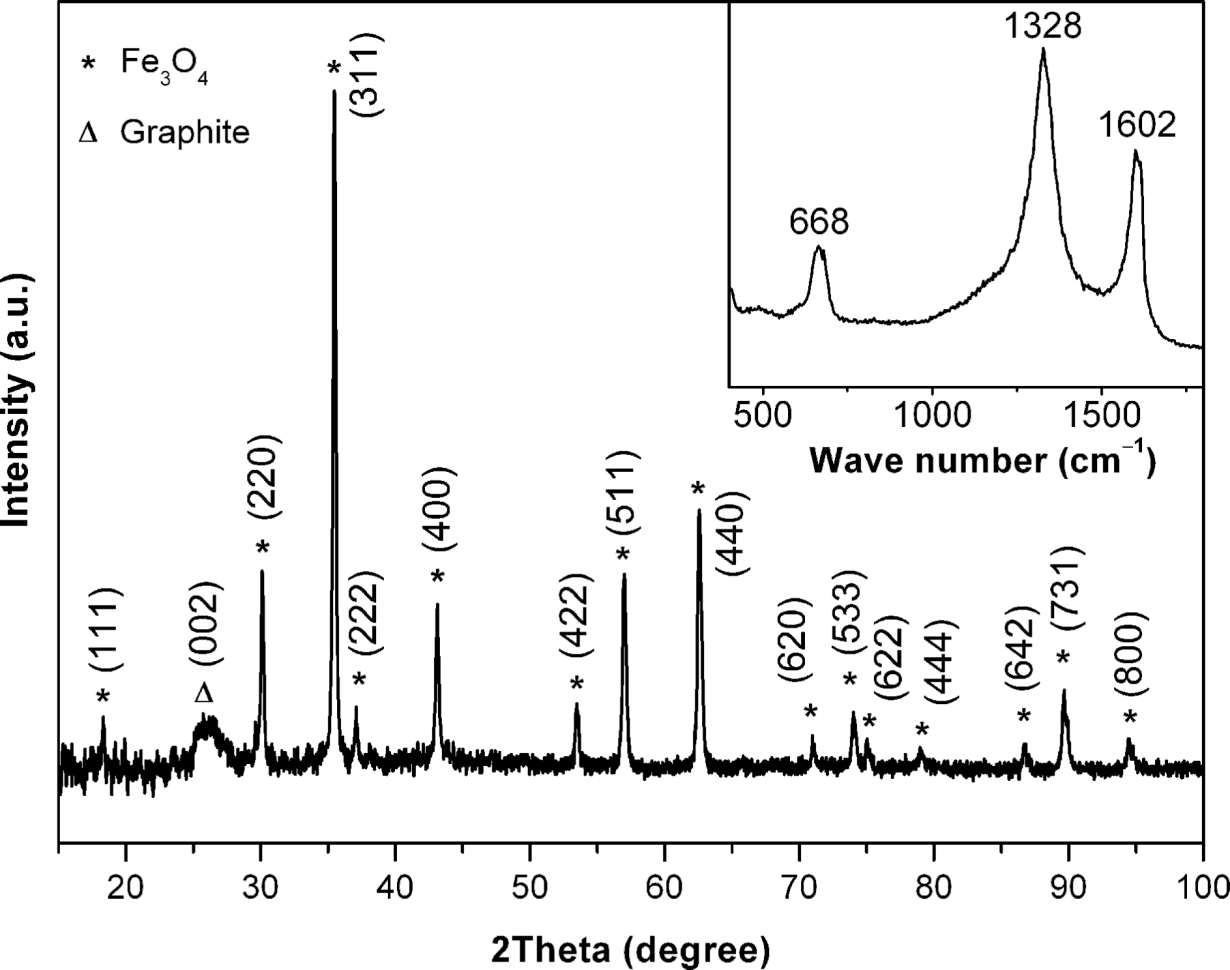
XRD pattern and Raman spectrum (inset) of [Fe_3_O_4_-C].

The X-ray powder diffraction (XRD) pattern and the Raman spectrum of the as prepared nanocomposite are shown in Figure 1. All the diffraction peaks can be attributed to two well-defined phases, which are hexagonal-phase graphitic carbon {26.4° (002); JCPDS-041-1487} and cubic-phase Fe_3_O_4_ {JCPDS-019-0629}. No signals for metallic iron or other oxides were detected in the XRD pattern, which indicates that the oxidation reaction was selective, and formed exclusively Fe_3_O_4_. Scherrer analysis was performed on high intensity Bragg peaks (220, 311, 400, 511 and 440) of Fe_3_O_4_, and the mean crystallite size was calculated to be 14 nm. The Raman spectrum of the composite showed two bands at 1328 and 1602 cm^−1^, which are characteristic of the D (disorder-induced phonon mode [17]) and G (graphitic lattice mode *E*_2_*_g_* [18]) bands of carbon, respectively. The intensity ratio *I*_G_/*I*_D_ of 0.7 indicates that a significant quantity of disordered carbon is also present in the nanocomposite. In addition, the *A*_1_*_g_* vibration mode of the Fe_3_O_4_ peak appeared at 668 cm^−1^, which agrees well with the literature value for pure as well as for graphene encapsulated Fe_3_O_4_ [11,19]. The infrared spectrum of the nanocomposite exhibited a broad band at 560 cm^−1^, which is typical for the Fe−O vibration of Fe_3_O_4_ [20].

**Figure 2:**
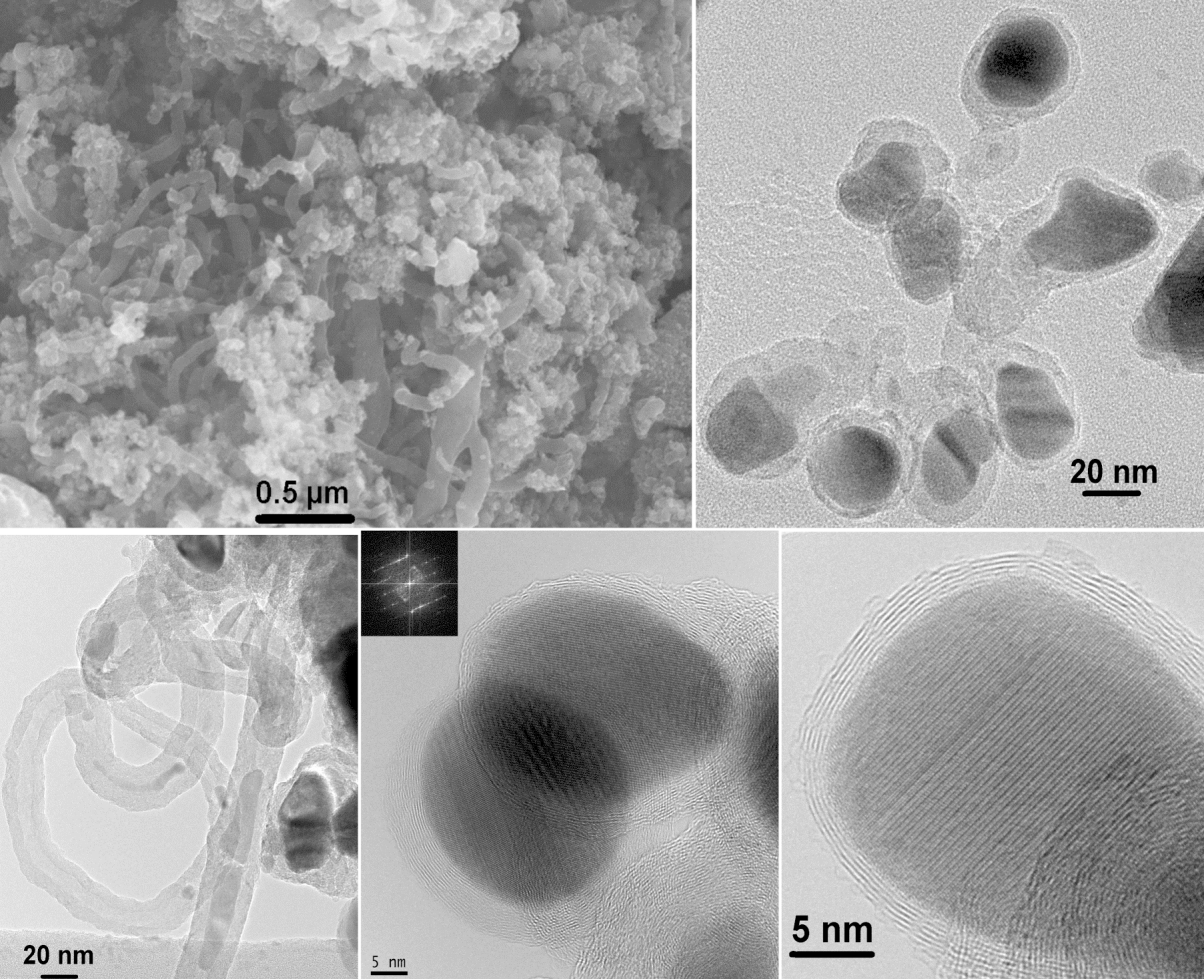
SEM (top left) and TEM images of [Fe_3_O_4_–C].

The SEM image of [Fe_3_O_4_–C] (Figure 2) shows that the material consists of interlinked nanotubes and nanogranular structures. The diameters of the tubes were in the range between 10 and 100 nm and their lengths varied up to several micrometers. A large number of tubes were encapsulated with Fe_3_O_4_ nanoparticles at their tips. However in some longer tubes, the particles were embedded in several places within the tube. TEM images of the nanogranular region of the composite confirmed the presence of a core–shell structure, containing Fe_3_O_4_ cores and graphitic onions shells. The interface between graphitic carbon and Fe_3_O_4_ with short-range disordered layers could be observed. The Fe_3_O_4_ particles were surrounded by roughly eight layers of graphite with an average carbon-coating thickness of about 3 nm. However, a few Fe_3_O_4_ particles were covered by several layers of carbon. In addition, a few incompletely/defectively carbon-coated as well as bare Fe_3_O_4_ nanoparticles could also be observed (Figure S2 in Supporting Information File 1). Fast Fourier transform (FFT) analysis of various HRTEM images (of crystallites located inside or outside of carbon shells, see Figure S3 in Supporting Information File 1) reveal that the observed lattice spacings fit very well to the cubic Fe_3_O_4_ (space group *Fd*3*m*) detected by XRD. The encapsulated Fe_3_O_4_ particles have diameters in the order of 30 nm. The lattice spacing of the adjacent graphitic layers is typically around 0.36 nm.

**Figure 3:**
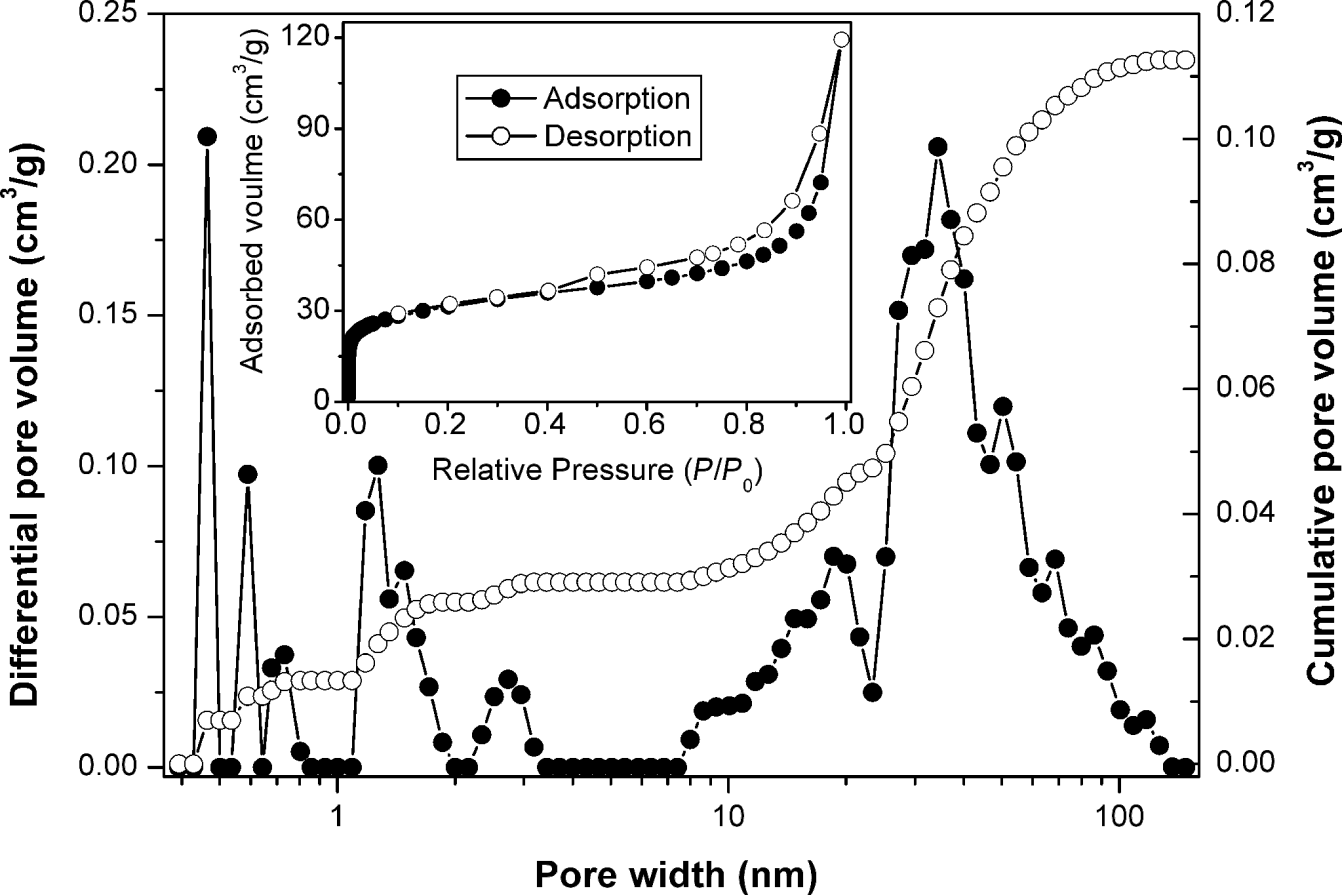
Nitrogen isotherm (inset) and pore width profile (cumulative: open circles, differential: filled circles) of [Fe_3_O_4_–C] measured at −196 °C.

The nitrogen adsorption–desorption measurement shows type-IV isotherms with an H_3_-type hysteresis loop (Figure 3), which indicates a mesoporous (pore width < 50 nm) nature of the material [21]. In addition, a sharp increase of the adsorbed gas at very low relative-pressures suggesting the presence of micropores (pore width < 2 nm). The micro- and mesoporous volumes were determined as 0.026 and 0.07 cm^3^·g^−1^, respectively. The differential pore volume estimated from the adsorption branch of isotherm by using the DFT model [22] suggests that the mesopore sizes were distributed between 10 and 35 nm. The BET specific surface area was calculated to be as high as 110.6 m^2^·g^−1^. It has been found that porous electrode materials can facilitate the diffusion of Li ions to active sites with less resistance and can also withstand the change of volume during the charge/discharge cycling [23]. Thus, the micro- and mesopores of [Fe_3_O_4_–C] could act as buffer for the volume change during redox cycle, which would lead to an enhanced cyclic performance as an anode material for LIBs.

**Figure 4:**
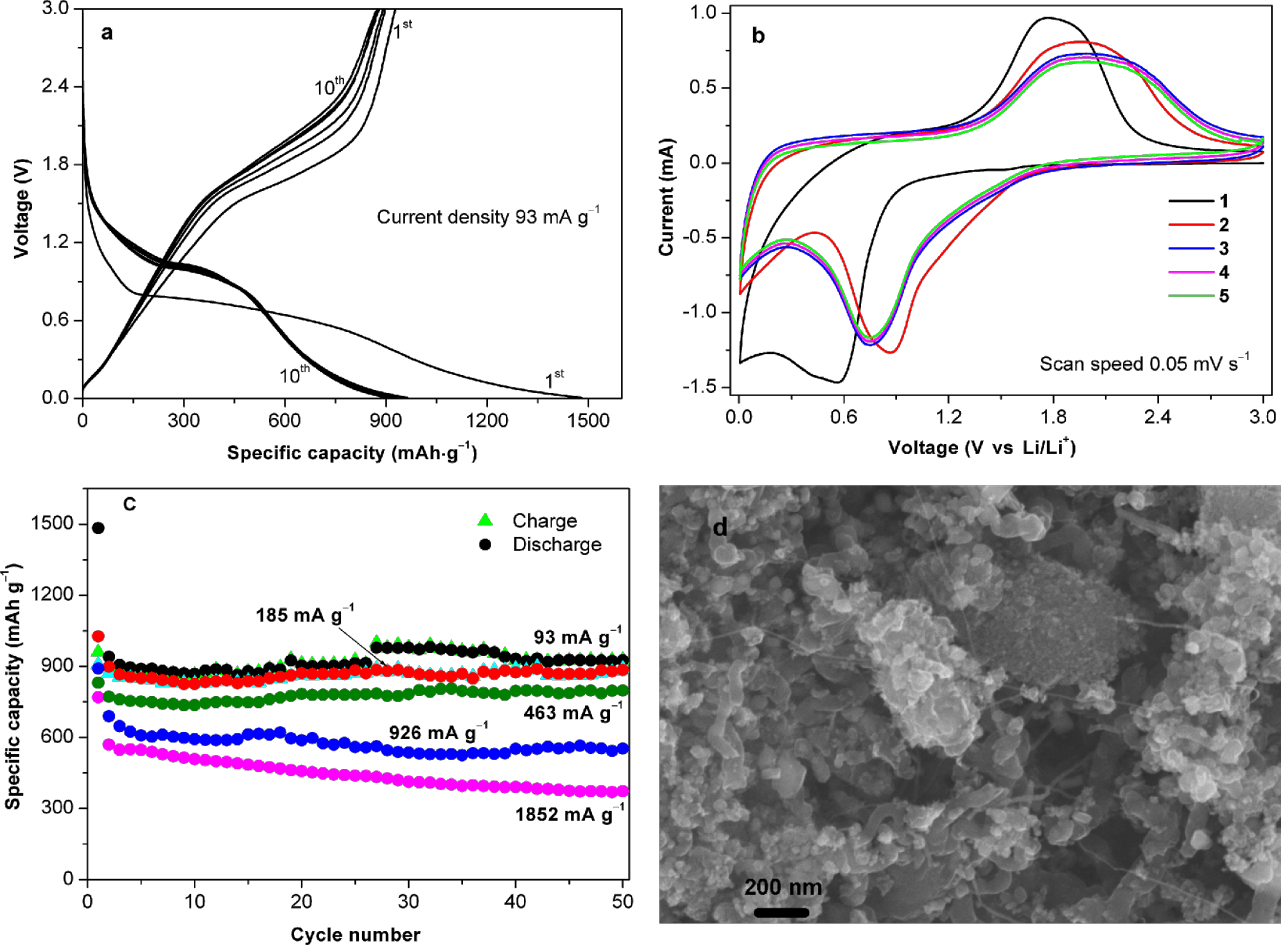
Electrochemical properties of [Fe_3_O_4_–C]. (a) Charge/discharge curves, (b) cyclic voltammograms, (c) rate performance profile, and (d) SEM image of electrode after 50 cycles at 93 mA·g^−1^.

The electrochemical performance of the [Fe_3_O_4_–C] anode has been evaluated with respect to Li metal. Figure 4a shows a typical galvanostatic profile for a [Fe_3_O_4_–C] cell cycled between 3.0 and 0.005 V at 93 mA·g^−1^. The obtained charge/discharge profiles are comparable to those of various Fe_3_O_4_ electrodes tested at similar current and voltage ranges [7–11]. During the first discharge the potential dropped abruptly down to about 0.8 V, which can be ascribed to the reaction of {Fe_3_O_4_ + *x*Li → Li*_x_*Fe_3_O_4_} [24]. The long plateau corresponds to the conversion reaction and the sloping part of the discharge curve can be assigned to the formation of the solid electrolyte interface (SEI) layer, as well as to the formation of a gel-like film through the reaction of Fe^0^ and electrolyte [7–11]. The electrode exhibits a first-discharge capacity of 1480 mAh·g^−1^ (based on the composite weight) and a first-charge capacity of 960 mAh·g^−1^. The capacity decreases marginally over the first few cycles and then stabilizes at about 920 mAh·g^−1^ in the subsequent 50 cycles. The coulombic efficiency after the first cycle remained at nearly 100%. The cyclic voltammogram of [Fe_3_O_4_–C] is comparable to that of other Fe_3_O_4_ electrodes [9–11], and shows a cathodic wave at 0.56 V and an anodic wave at 1.78 V, which correspond to the Fe^3+^/Fe^2+^-to-Fe^0^ redox couple. The irreversible wave at 0.4 V can be ascribed to the formation of the SEI. In the subsequent cycles, the reversible waves shifted slightly to more positive potentials. The CV curves of three successive scans almost overlap which reveals the good reversibility of the composite electrode.

Besides the cyclic stability, the electrode also exhibits a moderate rate capability performance. At current densities below 926 mA·g^−1^ the electrode exhibited good cyclic performances and an excellent capacity retention. When the electrode was cycled at 1852 and 2780 mA·g^−1^, respectively, the observed capacity retentions between the 3rd and the 50th cycles were about 72 and 63%, respectively. A similar trend has also been observed in other Fe_3_O_4_/C systems at high current rates [9,11], which could be ascribed to the slow conversion reaction kinetics. SEM images of the electrode cycled for 50 cycles at 93 mA·g^−1^ show a morphology similar to that of the original composite. This indicates that the active materials remain intact during cycling.

Both Fe_3_O_4_ and carbon are electrochemically active components for Li-ion storage and contribute to the overall capacity of the electrode. The good electrochemical performance of the composite can thus be attributed to its special morphology, porosity and also the synergistic effect by combining metal oxide and carbon nanotubes, which provides better electronic and ionic transport, as well as a tolerance toward the volume change during the reaction.

In summary, a new carbon encapsulated Fe_3_O_4_ nanocomposite was synthesized by a simple one-step pyrolysis of Fe(CO)_5_. The nanocomposite exhibits well-constructed core–shell and nanotube structures with Fe_3_O_4_ cores and graphitic shells/tubes. The nanocomposite electrode exhibits a stable reversible capacity of 920 mAh·g^−1^ at 93 mA·g^−1^ in the subsequent 50 cycles. Further experiments are underway to check its extended stability and capacity retention behaviour. We believe that this method opens a simple way for producing carbon encapsulated metal oxide nanocomposites for energy storage, catalysis, and magnetic applications.

## Supporting Information

File 1 General procedures and additional figures.
