# Supplementary material for: A facile synthesis of a carbon-encapsulated Fe3O4 nanocomposite and its performance as anode in lithium-ion batteries
Source: Beilstein J Nanotechnol. 2013 Oct 30;4:699–704. doi: 10.3762/bjnano.4.79 (PMC3817607; doi:10.3762/bjnano.4.79)
Supplement: File 1 — General procedures and additional figures. [file Beilstein_J_Nanotechnol-04-699-s001.pdf]

Supporting Information

for

Facile synthesis of a carbon-encapsulated Fe_3O_4 nanocomposite and its performance as anode in lithium-ion batteries

Raju Prakash[1,2], Katharina Fanselau[1], Shuhua Ren[1], Tapan Kumar Mandal[1,3], Christian Kübel[4], Horst Hahn[1] and Maximilian Fichtner*[1,5]

Address: [1]Institute for Nanotechnology (INT), Karlsruhe Insititute of Technology (KIT), Hermann-von-Helmholtz-Platz 1, Karlsruhe, 76344, Germany, [2]current address: Centre for Automotive Energy Materials (CAEM), International Advanced Research Centre for Powder Metallurgy and New Materials (ARCI), Taramani, Chennai-600113, India, [3]Faculty of Science and Technology, ICFAI University, Selaqui, Dehradun-248197, India, [4]Karlsruhe Nano Micro Facility (KNMF), Karlsruhe Institute of Technology (KIT), Hermann-von-Helmholtz-Platz 1, Karlsruhe, 76344, Germany and [5]Helmholtz Institute Ulm (HIU), Albert-Einstein-Allee 11, Ulm, 89081, Germany

Email: Maximilian Fichtner* - m.fichtner@kit.edu

* Corresponding author

General procedures and additional figures

Experimental

All operations were carried out in an Ar-filled glove box and/or by using standard Schlenk techniques. Iron pentacarbonyl (99.999%) purchased form Aldrich Co. was used as received.

Synthesis of [Fe_3O_4-C]: $Fe(CO)_5$ (1.0 g, 5.1 mmol) was sealed into a closed stainless steel Swagelok reactor (SS-8-WVCR-6-DF; inner diameter, 10.2 mm; length 46.7 mm) containing vacuum coupling radiation (VCR) type of fittings on both ends. The pyrolysis was performed in a home-made designed rotating furnace setup as described previously (see Prakash et al. *J. Power Sources*, 2011, **196**, 5936). The quartz tube (inner diameter 5 cm, length 75 cm) was placed horizontally inside the tube furnace (GERO) without touching the ceramic wall of the furnace. Both ends of the tube were supported by wheels mounted on top of the Y-shaped rods, while the bottom of the rods connected to an adjustable base plate. One of the wheels was coupled to a motor (DOGA gear motor, 12 V/DC, 25 rpm, 6 Nm). The reactor was placed in the center of the tube and fixed on both sides with a quartz mat. The tube was rotated continuously at a frequency of 10 rpm. Then, the furnace was heated to 730 °C (the temperature measured at the reactor was 700 °C) at a temperature ramp of 5 $°C \cdot min^{-1}$ and kept at that temperature for 3 h. Then the reactor was allowed to cool down to room temperature naturally. Due to various gaseous materials produced during pyrolysis, pressure developed inside the reactor. It was opened carefully inside a glove box, and a fine black powder produced was collected (Yield: 360 mg, 91% with respect to Fe). Elemental analysis, C 30%; XRD 2θ (hkl): 18.3 (111), 30.1 (220), 35.4 (311), 37.1 (222), 43.1 (400), 53.4 (422), 57.0 (511), 62.5 (440), 70.9 (620), 74.7 (533), 78.9 (444), 86.8 (642), 89.6 (731), 94.5 (800) for Fe_3O_4 (Ref: 00-019-0629); 26.4 (002) for graphitic carbon (Ref: 00-041-1487).

Characterization: The phase analysis of the nanocomposite was performed by using a Philips X'PERT diffractometer with Cu Kα radiation. PANalytical X'Pert Data Collector and X'Pert HighScore software were used for data acquisition and evaluation, respectively. The samples to be analyzed were spread onto a silicon single crystal in the glovebox and sealed with an airtight hood made of Kapton foil, which is out of the focus of the spectrometer. The patterns were recorded at 25 °C in a 2θ range between 10 and 90°. Raman spectra were obtained using a WiTec CRM200 confocal Raman microscope with a laser excitation at 633 nm (HeNe Laser). Measurements from several different spots were accumulated in one spectrum**.** The morphology and microstructure of the nanocomposite were investigated by using scanning electron microscopy (SEM; Leo-1530) and transmission electron microscopy (TEM; image corrected FEI Titan 80-300 operated at 300 kV in TEM mode and HAADF-STEM mode, equipped with a Gatan Imaging filter Tridiem 863 and an EDAX s-UTW EDX detector). The sample for TEM analysis was prepared by dispersing the nanocomposite in pentane. A droplet was placed onto a carbon film supported on a copper grid and allowed to dry for at least 2 h. The grid was sealed under Ar and reopened to put them into the TEM, thereby minimizing exposure to atmosphere to about a minute. The nitrogen physisorption isotherm was measured using a Micromeritics ASAP 2020 system at 77 K. Prior to measurement, the sample was degassed at 350 °C for 12 h. Pore size distributions (PSD) were calculated based on a DFT model assuming slit-shaped pores. The specific surface area was determined according to the BET theory. Electrochemical properties of the nanocomposites as anode materials in lithium-ion cells were tested by the galvanostatic charge–discharge technique under the use of two-electrode Swagelok-type cells. The test electrodes consisted of the nanocomposite, carbon black and poly(vinyldifluoride-co-hexafluoropropylene) (SOLEF 21216/1001) at a weight ratio of

82:9:9. The mixture was wetted by several drops of NMP (*N*-methylpyrrolidone) and continuously mixed in a mortar for 10 min to form a homogeneous slurry. Then the slurry was coated on stainless steel disc (diameter 10 or 12 mm, thickness 0.3 mm) current collectors and the discs were allowed to dry (60 °C for 5 h and then 120 °C for 12 h) to form the test electrodes. The discs were weighed individually before and after coating of the electrode material to know the exact quantity of the material deposited on each disk. The test cells were prepared by using the coated nanocomposite as working electrode, metallic lithium (Goodfellow) as reference/counter electrode, glass fiber (GF/D; Whatman) as separator, and 1 M $LiPF_6$ in ethylene carbonate (EC) and dimethyl carbonate (DMC) (1:1 v/v) as the electrolyte. The galvanostatic charge/discharge cycling was performed in the potential range of 3.0 to 0.005V (vs. Li/Li^+) at a given constant current density by using a battery testing system (Arbin Instruments, BT2000 multi-channel system). The specific charge/discharge capacities were calculated based on the total amount of composite including the mass of the graphitic carbon. Cyclic voltammograms were performed by using an Autolab Potentiostat/Galvanostat 100 at a scan rate of 0.05 $mV \cdot s^{-1}$.

Figures

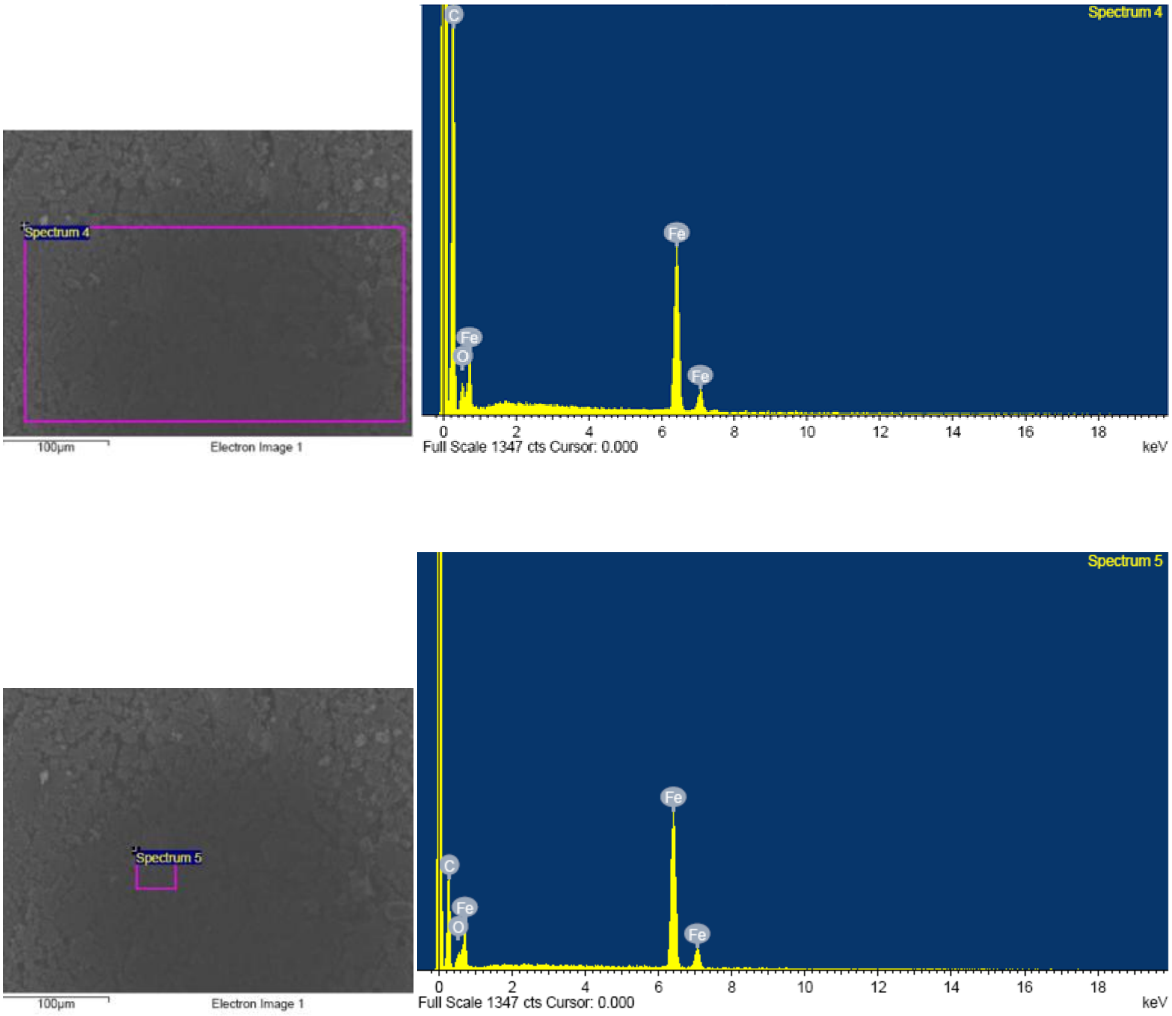

Figure S1: EDX elemental analysis of [Fe_3O_4-C] performed at various regions showing only the elements carbon, iron and oxygen. The weight ratio of Fe/O in all cases is ≈2.7 (theoretical value 2.62).

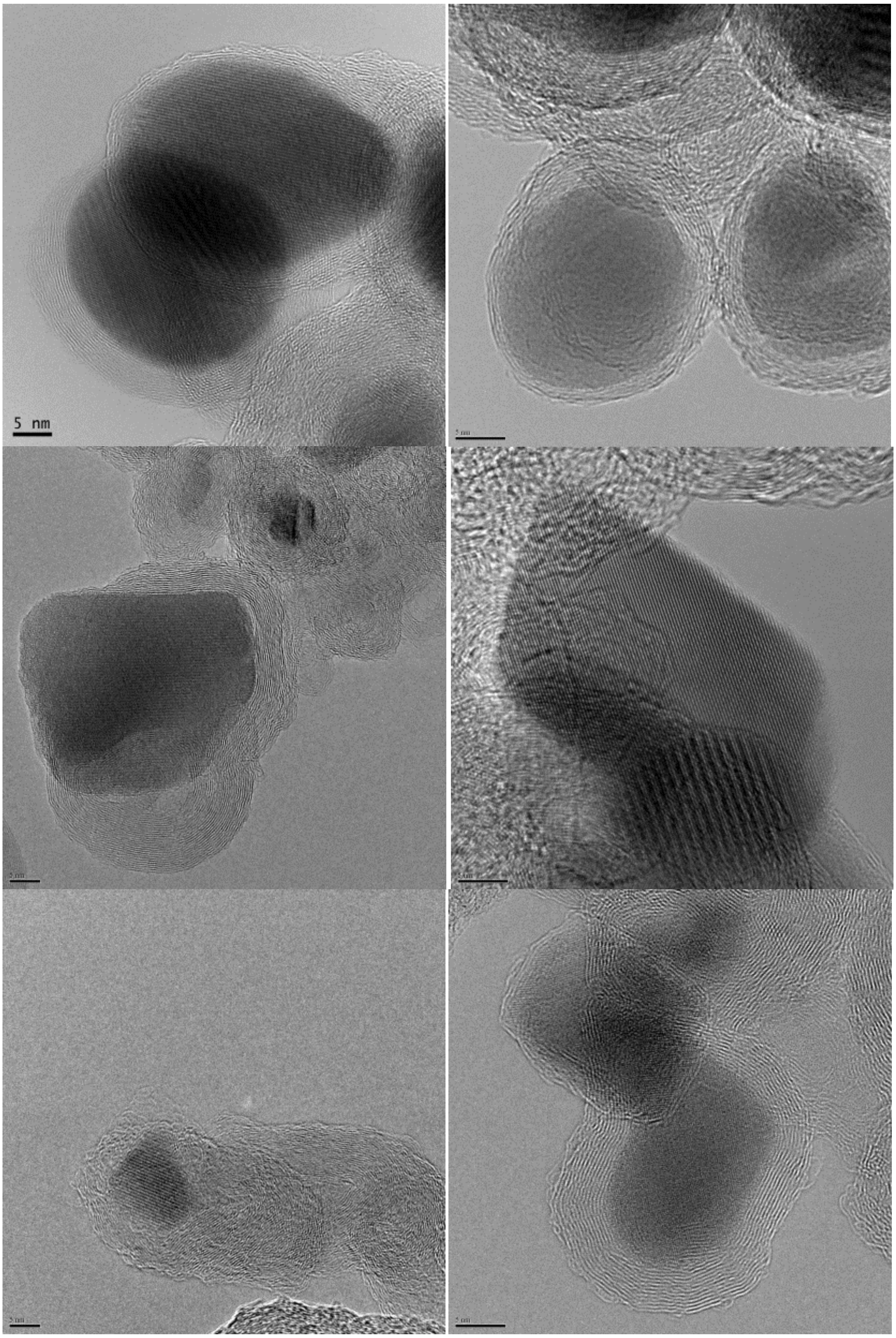

Figure S2: Selected HRTEM images of as-prepared [Fe_3O_4-C], which contain inhomogeneous, partial and excessive carbon coated, as well as uncoated Fe_3O_4 nanoparticles.

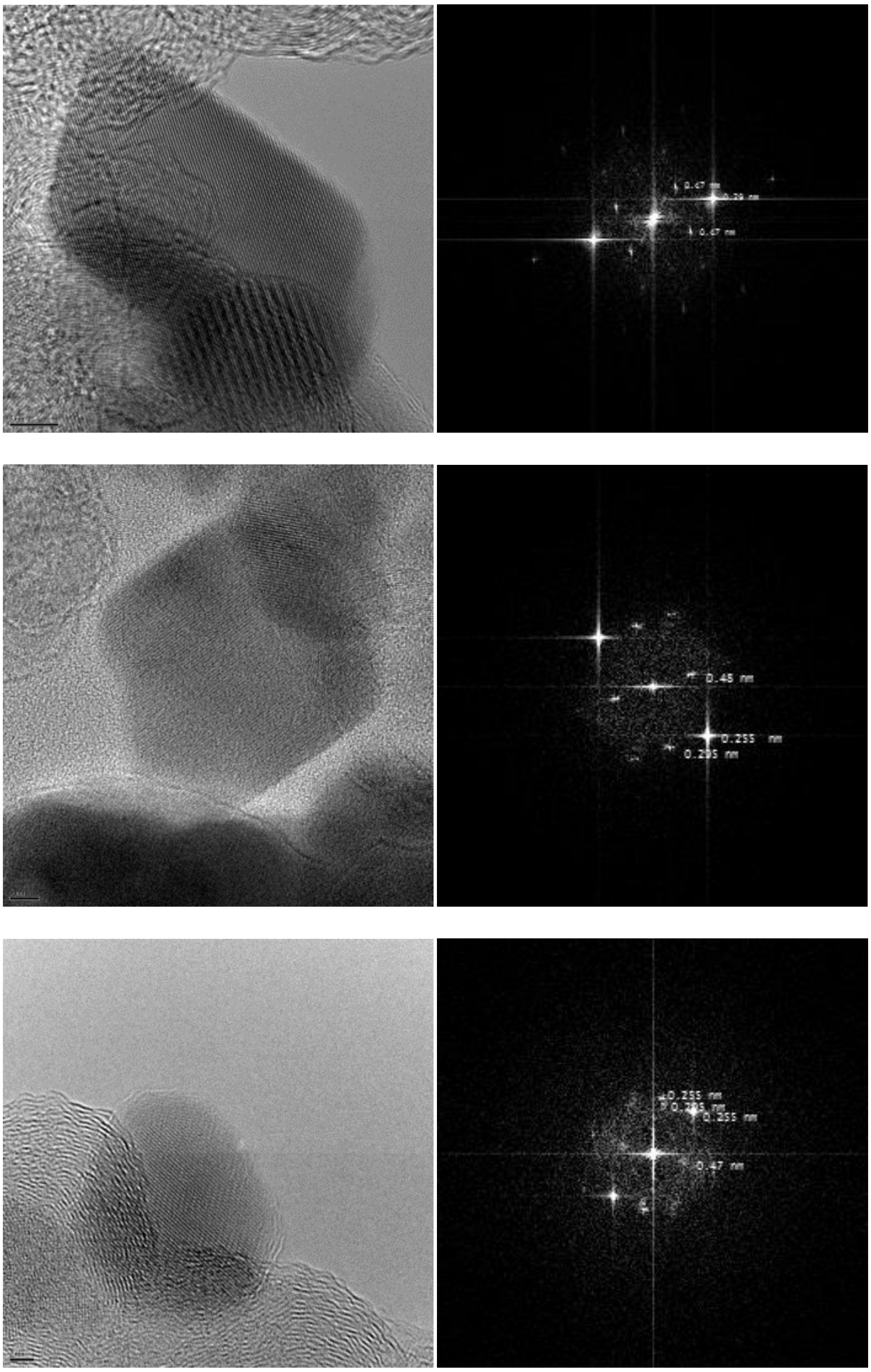

Figure S3: HRTEM images of [Fe_3O_4-C] showing some uncoated as well as partially coated Fe_3O_4 nanocrystals and their corresponding FFT images at zone axis 110 or 112.
